# Supplementary material for: Multiplexed single-cell transcriptional response profiling to define cancer vulnerabilities and therapeutic mechanism of action
Source: Nat Commun. 2020 Aug 27;11:4296. doi: 10.1038/s41467-020-17440-w (PMC7453022; doi:10.1038/s41467-020-17440-w)
Supplement: Supplementary file 1 — Supplementary Information [file 41467_2020_17440_MOESM1_ESM.pdf]

Multiplexed single-cell transcriptional response profiling to define cancer vulnerabilities and therapeutic mechanism of action

McFarland and Paolella et al.

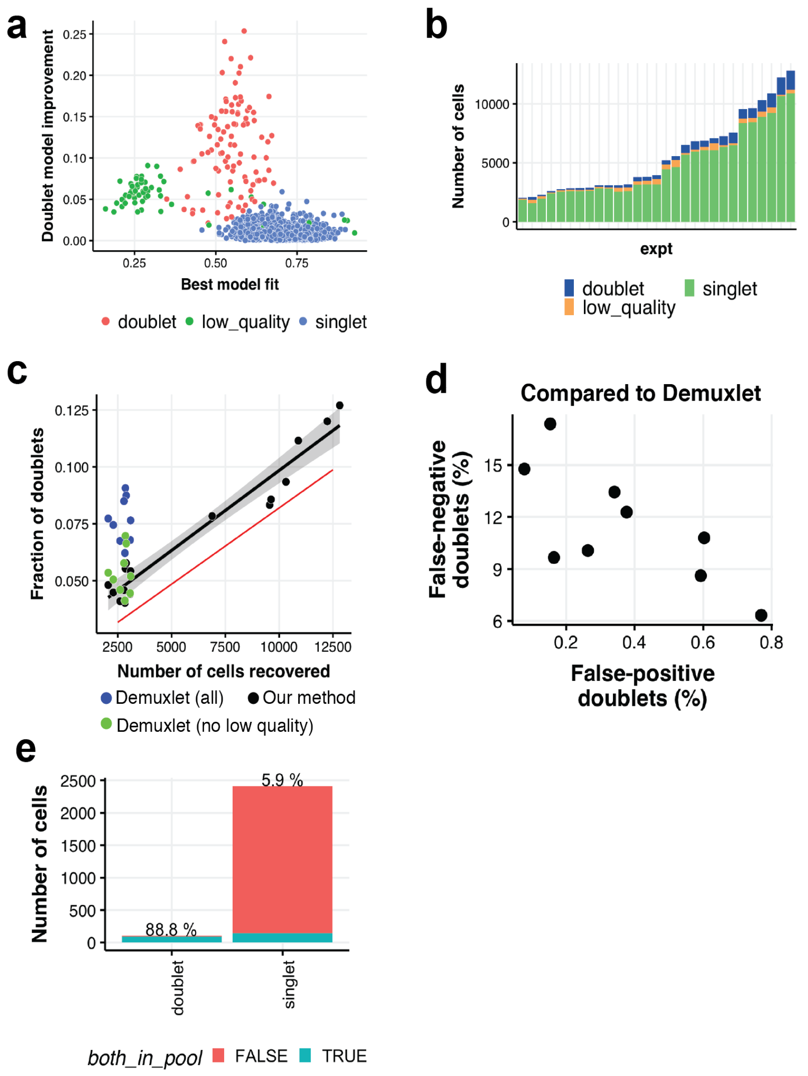

Supplementary Fig. 1: SNP-based detection of doublets and low-quality cells.

a) Scatterplot of parameters from the SNP-based models (see Methods) from an example experiment, showing classification of cells as singlets, doublets, and ‘low-quality’. The y-axis shows the improvement of the doublet model fit over the singlet model, while the x-axis shows the best model goodness-of-fit. **b)** Distribution of cells classified in each category across experiments. **c)** Proportion of cells classified as doublets in each experiment (excluding low-quality cells), as a function of the number of cells recovered in the experiment. Red line shows the trend-line from (Zheng et al., 2017) depicting the expected relationship of doublet probability with cell density. The back line shows the linear regression trend line (with the gray shaded region showing the 95% CI interval). Green and blue points show doublet proportions estimated using Demuxlet (Kang et al. 2018) (only for experiments with smaller pools), with and without exclusion of low-quality cells (based on our analysis pipeline) respectively. **d)** Using the Demuxlet doublet detection as ‘ground truth’, false-negative and false-positive rates for our doublet classification procedure are shown for each experiment. Overall, Demuxlet tended to produce a somewhat higher rate of detected doublets, even though both methods tended to call doublets at a higher rate than expected based on the cell loading density (**c**). **e)** For an example experiment, we estimated the proportion of cells where the most likely *doublet pair* of reference cell lines were both among the ‘in-pool’ cell lines (24/494 possible cell lines). 89% of cells classified as doublets had both identified reference cell lines among those in the experimental pool (n = 107 cells). For cells classified as singlets, there were only ~6% (approximately chance level) (n = 2417 cells).

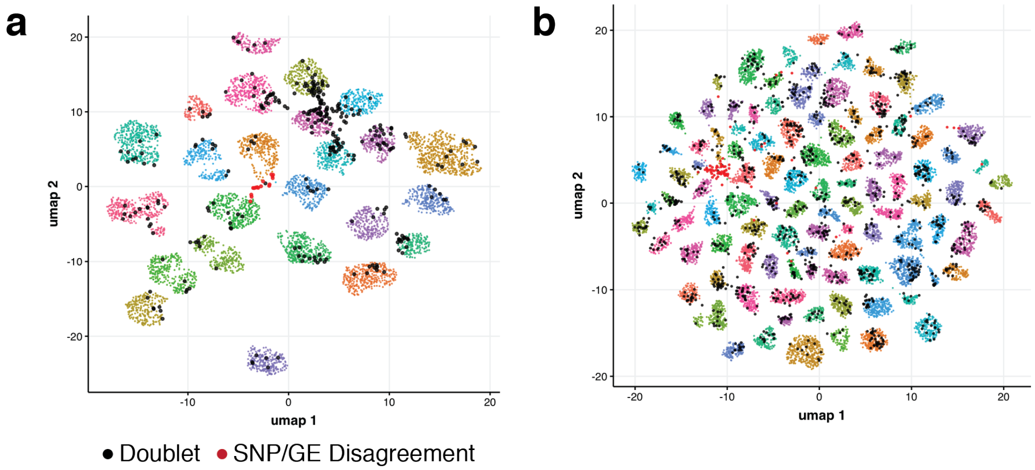

Supplementary Fig. 2: Agreement between SNP-based and GE-based cell classification.
a) UMAP representation of expression profiles from an example negative-control experiment (combination of DMSO-treated and untreated cells) in a pool of 24 cell lines. Cells show clear clustering by cell line (color indicates SNP-based parental cell line classification). Black dots show cells classified as doublets. Red dots indicate cells where gene expression and SNP-based classifications disagree (0.2% of single cells, 16/6926). **b)** Same as **a**, for an example (DMSO-treated) dataset from a 99 cell-line pool. Gene expression and SNP-based classifications disagreed for only 0.05% of single cells.

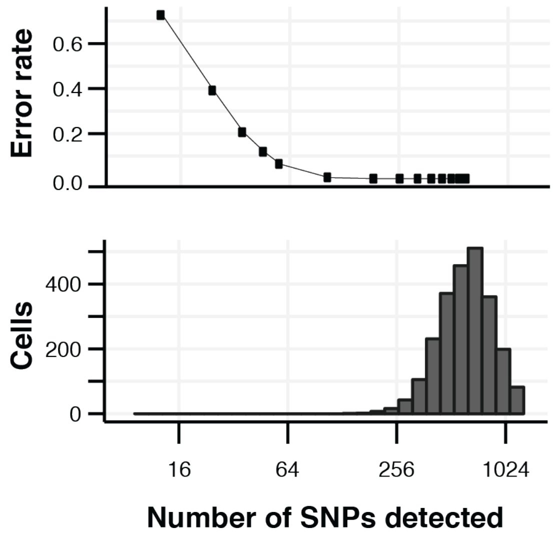

Supplementary Fig. 3: Dependence of SNP-based cell classification on number of detected SNPs
(*Top*) Error rate of cell classification was estimated based on the fraction of cells (n = 2,417 cells) classified among those in the experimental pool (24/494 reference cell lines from this example dataset). Classification accuracy for cells with fewer SNP sites detected was estimated by randomly down-sampling the single-cell SNP reads. (*Bottom*) Distribution of the number of SNP sites detected for the cells (n = 2,417 cells) measured in the example experiment.

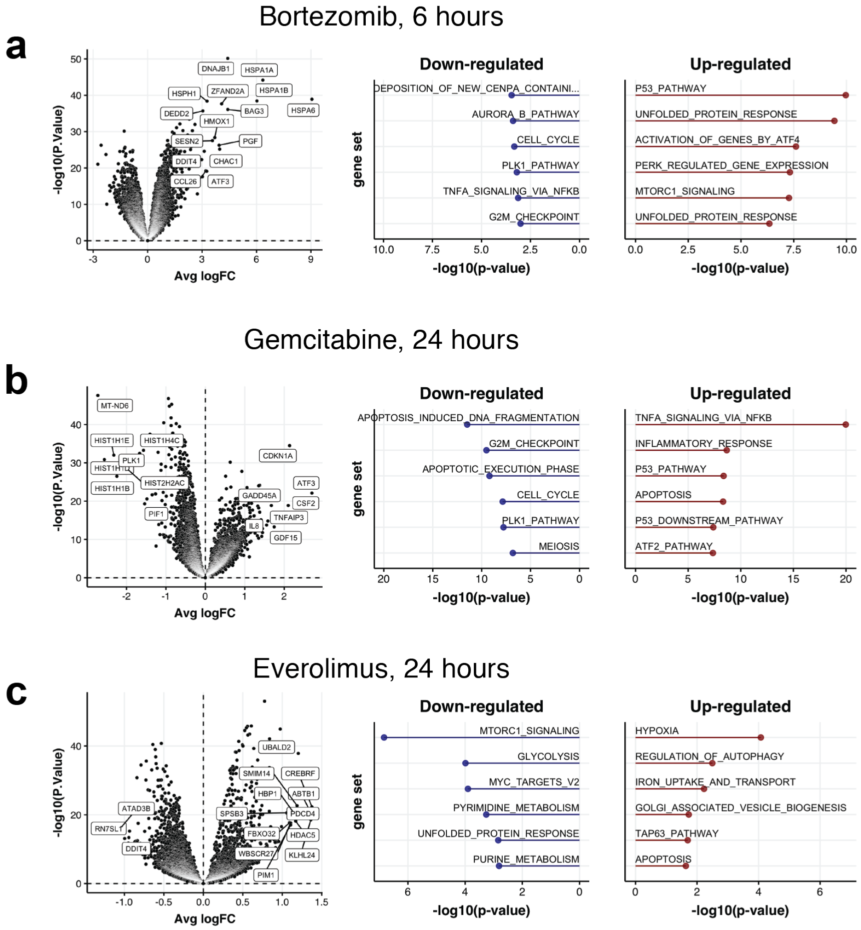

Supplementary Fig. 4: Identification of compound MOA from transcriptional response profiles.

a) (Left) Volcano plot of the (across-cell-line) average transcriptional response to bortezomib (6 hours post-treatment). Differential expression analyses were performed using the limma-trend pipeline (Law et al. 2014; Ritchie et al. 2015). (Right) Top gene sets enriched among the most strongly up- and down-regulated genes.. **b)** Same as a for gemcitabine treatment (24 hours post-treatment). **c)** Same as **a-b**, for everolimus treatment (24 hours post-treatment).

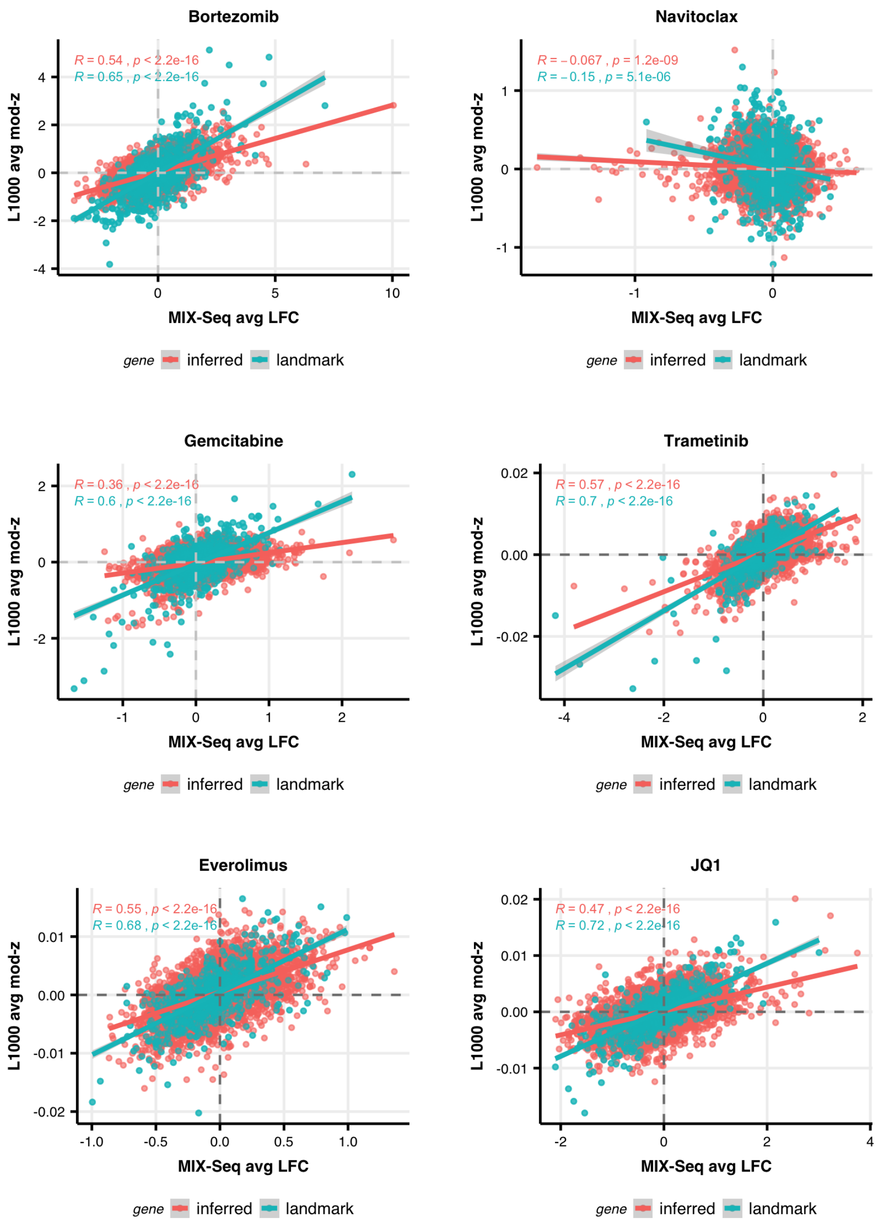

Supplementary Fig. 5: Agreement between MIX-Seq and L1000 transcriptional response profiles.

Comparison of average response profiles measured using MIX-Seq and the L1000 platform (Subramanian et al. 2017) for six different compounds. Blue dots represent the 978 'landmark' genes measured directly in the L1000 platform, while the red dots represent computationally inferred genes in the L1000 dataset. In each case responses are averaged across available cell lines. L1000 data were also averaged across available doses, and time points to simplify comparisons (Methods). For the MIX-Seq data, bortezomib and trametinib responses were measured at 24 hours post-treatment. Five of the six drugs show good agreement in the average response profiles. Agreement was poor for navitoclax, though we did not observe a robust transcriptional response to navitoclax in the MIX-Seq data. Pearson correlation coefficients, and associated p-values, are reported in each plot for the landmark genes and all genes in blue and red respectively.

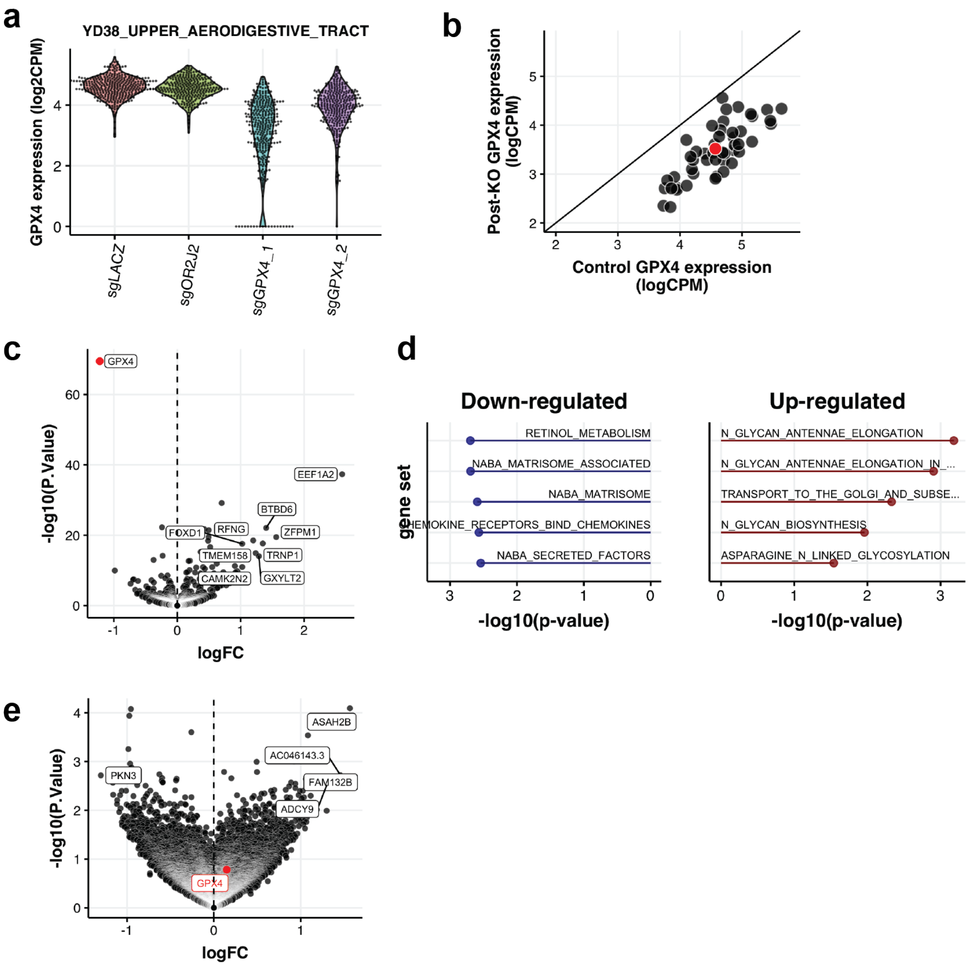

Supplementary Fig. 6: Pooled transcriptional profiling of genetic perturbation.

a) Distribution of expression levels of GPX4 in an example cell line for cells infected with two sgRNAs targeting GPX4, vs. two control sgRNAs (sgLACZ: non-targeting control, sgOR2J2: ‘cutting control’), showing robust on-target knockdown of GPX4 expression. **b)** Comparison of average GPX4 expression across pool of 50 cell lines following GPX4 KO compared with control, showing consistent on-target KD across cell lines. Red dot shows example cell line from **a**. **c)** Volcano plot showing average transcriptional response to GPX4 KO across all cell lines. The top up-regulated gene EEF1A2 has been shown to play a role in regulating lipid metabolism (Jeganathan and Lee 2007), consistent with the role of GPX4 in lipid metabolism. **d)** Gene set analysis of the average GPX4 KO response showed up-regulation of N-glycan synthesis, which has been reported to regulate glutathione levels (Calle et al. 2000). **e)** Volcano plot comparing response to GPX4 KO in GPX4-dependent (n=18) vs. non-dependent (n=15) cell lines (see Methods). No genes were found to be significantly differentially expressed between the groups (at an FDR threshold of 0.1).

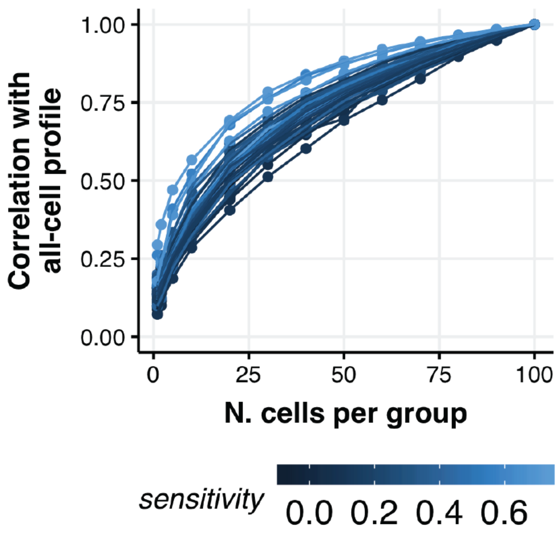

Supplementary Fig. 7: Impact of cell population size on estimates of transcriptional response profiles.

To understand how estimates of each cell line's transcriptional response depended on the number of cells sampled per treatment condition we performed a downsampling analysis. Specifically, for measured trametinib response (24 hours post-treatment, as in **Fig. 2**), we took the set of cell lines which had at least 100 cells sampled in each condition (n = 45), and restricted the data to a random set of 100 cells per condition. We then estimated the LFC transcriptional response profile of each cell line using random subsets of cells and compared these subsampled estimates with the profiles derived from the starting set of 100 cells per condition. Profile similarity was assessed by the Pearson correlation of LFC vectors across the 5000 most variably-expressed genes, averaged across 5 repetitions of the downsampling procedure for each cell line. Each line represents data from a different cell line, colored by the cell line's measured trametinib sensitivity (1 – AUC).

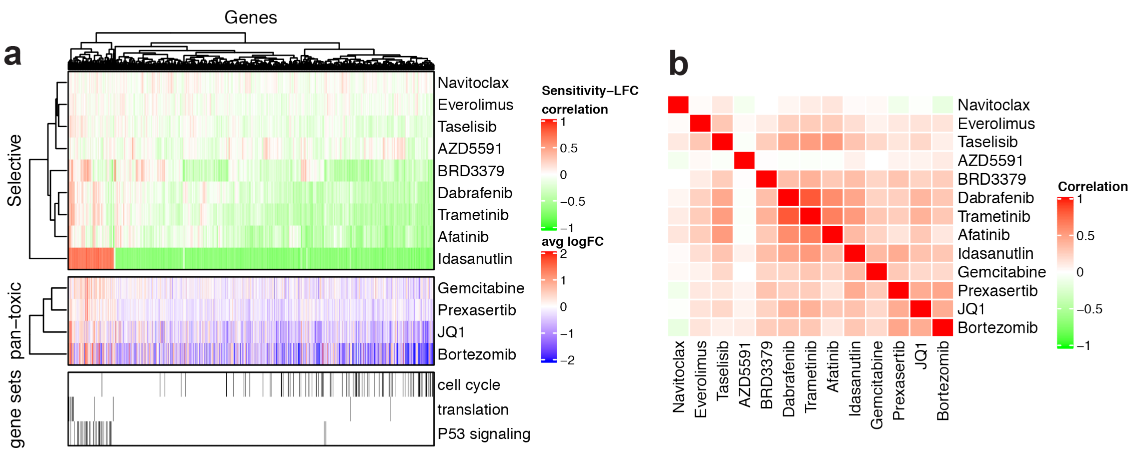

Supplementary Fig. 8: Similarity of viability-related transcriptional responses across drugs. **a)** (Top) Heatmap of correlation between transcriptional response and drug sensitivity across cell lines for the 1000 genes with the strongest average correlation for the 8 selective drugs. (Middle) Heatmap of average logFC drug response across cell lines for 4 pan-toxic drugs tested showing similarity to the viability-related response profiles above. (Bottom) Depiction of gene membership in 3 MSigDB gene sets (cell cycle = HALLMARK_G2M_CHECKPOINT; translation = REACTOME_TRANSLATION, and P53 signaling = HALLMARK_P53_PATHWAY). **b)** Matrix of correlations for the transcriptional response profiles shown in **a** across all pairs of compounds. Viability-related responses of selective compounds, and average responses for pan-toxic compounds, were broadly similar across these 1000 genes (with the exception of navitoclax and AZD5591).

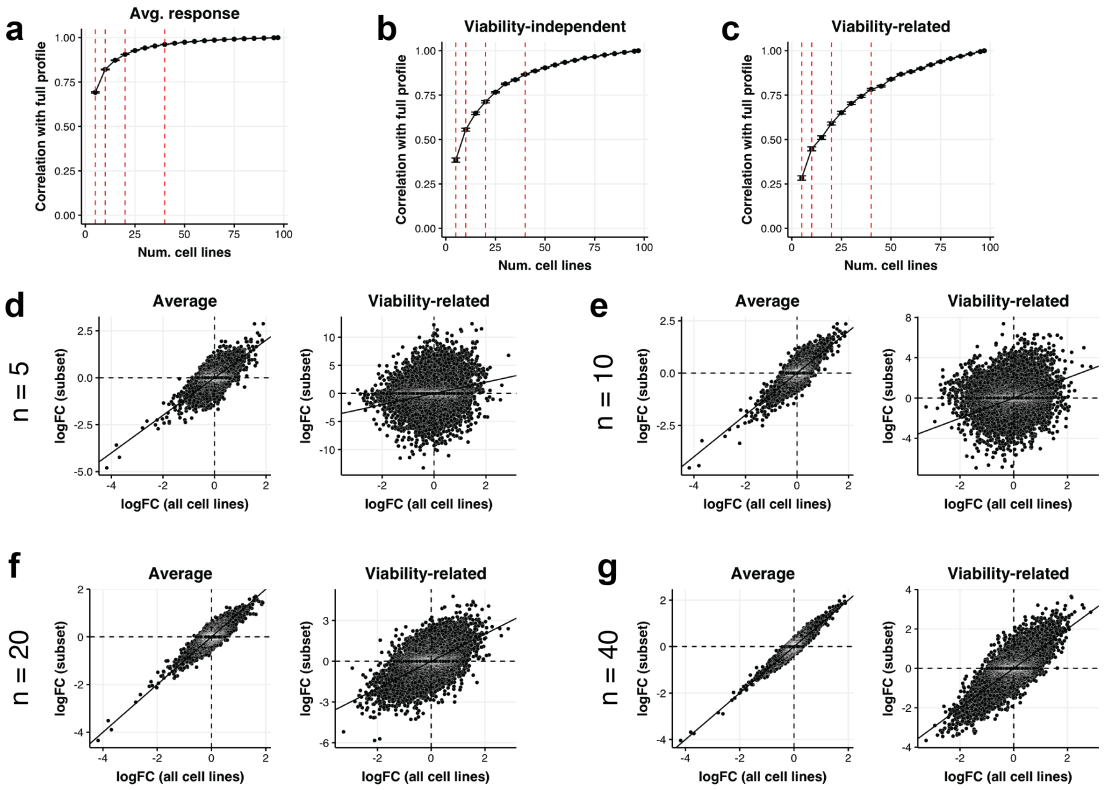

Supplementary Fig. 9: Effect of cell line sample size on estimation of transcriptional response components.

Analyses of the trametinib response components (as in **Fig. 2**; n = 99 cell lines) were repeated with random subsets of the cell lines. **a-c)** Average correlation between the estimated response profile (logFC) when using a subsample of cell lines vs. all the cell lines for the average (**a**), viability-related (**b**), and viability-independent (**c**) components respectively. Error bars show interval +/- s.e.m. Vertical red lines indicate the subsample sizes shown in **d-g**. **d-g)** Scatterplot comparisons of example estimates of average and viability-related response components using all cell lines vs. random subsets of increasing size, from 5 to 40. (d) n = 5 cell lines, (e) n = 10 cell lines, (f) n = 20 cell lines, (g) n = 40 cell lines.

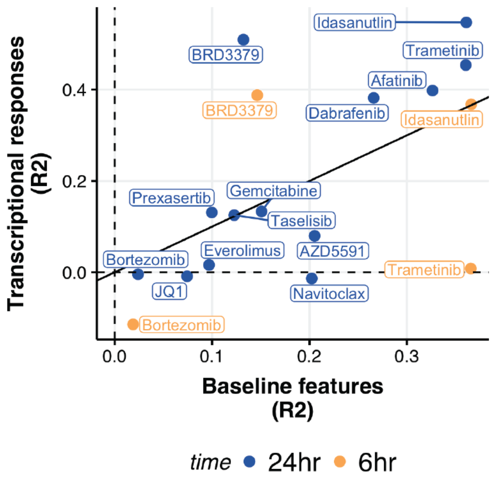

Supplementary Fig. 10: Comparison of predictive models using transcriptional responses vs. baseline features.

Same as **Fig. 3b**, but where the model trained on baseline features used data from all available cell lines (rather than using only the cell lines with measured transcriptional response data).

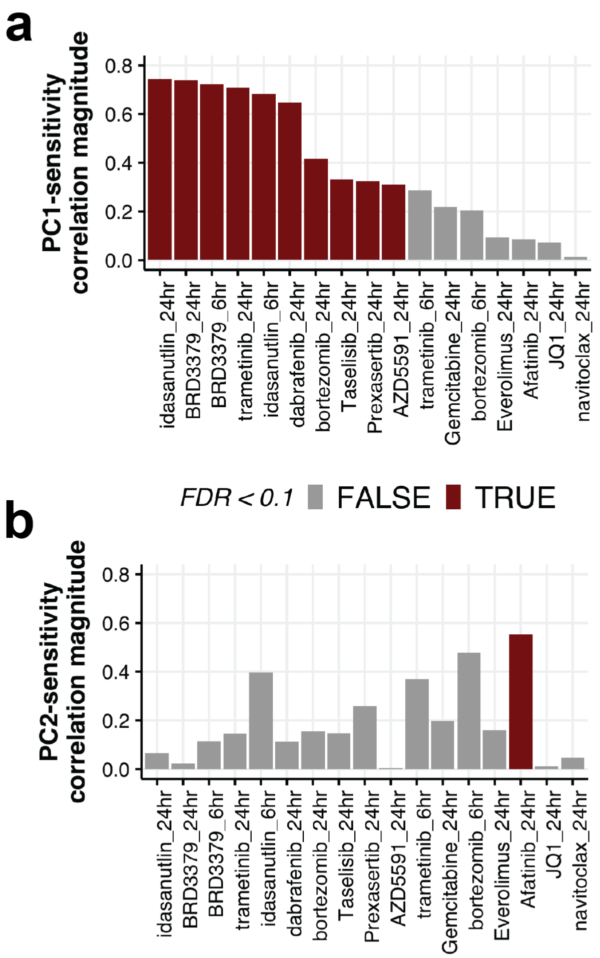

Supplementary Fig. 11: Top principle component of transcriptional responses is well correlated with variation in drug sensitivity.

Pearson correlation between each cell line's measured drug sensitivity and the projection of its transcriptional response profile onto the first (**a**) or second (**b**) principle component, computed for each treatment (drug and post-treatment time point). Significant correlations (FDR < 0.1) are shown in red. For a majority of experiments drug sensitivity is well captured by the first PC. For afatinib, PC2 captures the variation in drug sensitivity.

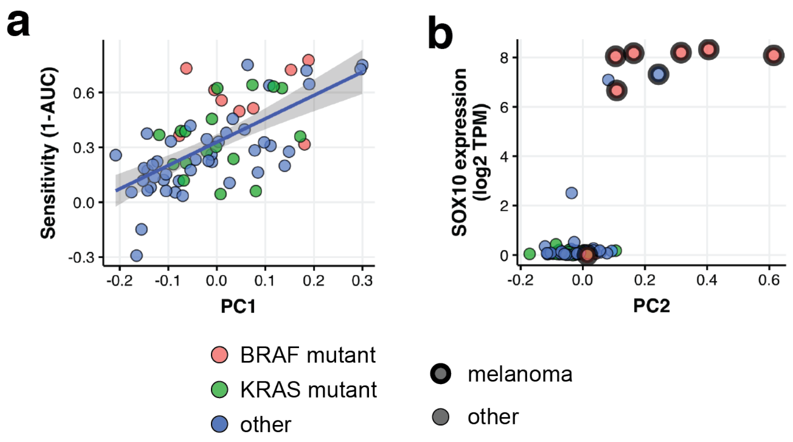

Supplementary Fig. 12: PCA identifies multiple components underlying variability in trametinib response.

a,b) Same as **Fig. 3e,f** for an additional experiment measuring trametinib responses (24 hours post-treatment) on a separate pool of 97 cell lines. **a**) The first PC was highly correlated with variation in trametinib sensitivity across cell lines. The blue line shows the linear regression trend line (with the 95% CI interval shown with the shaded gray). **b**) The second PC captured differences in the response among cell lines with high SOX10 expression (mostly BRAF mutant melanomas).

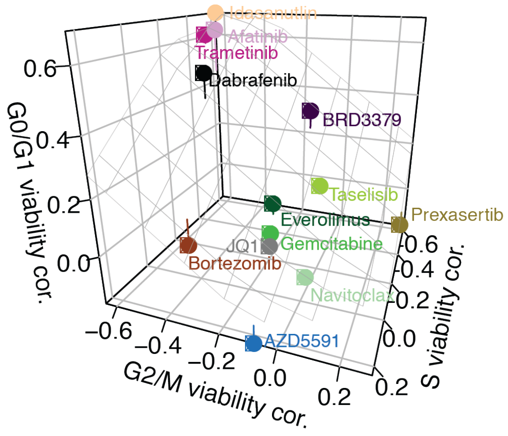

Supplementary Fig. 13: Drug-induced changes in cell cycle phase are well correlated with measured viability effects across cell lines.

Comparison of the Pearson correlation between measured drug sensitivity and the changes in G2/M-, G0/G1-, and S-phase cell proportions for all compounds. Lattice shows the regression plane of the z-coordinate.

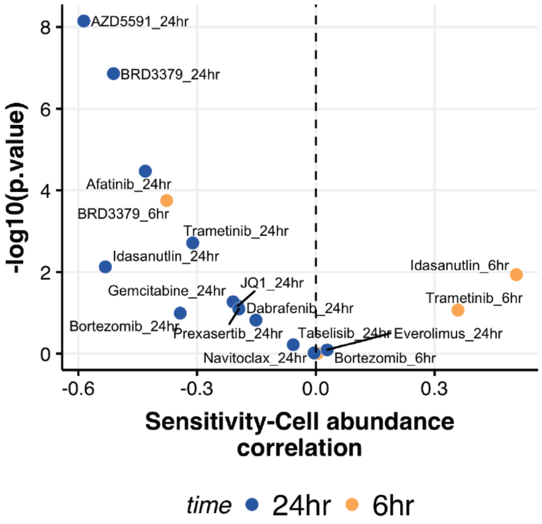

Supplementary Fig. 14: Direct measurement of relative drug sensitivity via scRNA-seq.

Correlation, and associated p-values, between existing drug sensitivity data (Iorio et al. 2016; Corsello et al. 2020) and the measured change in relative cell abundance (based on pooled scRNA-seq data). The time point of scRNA-seq profiling is indicated by dot color.

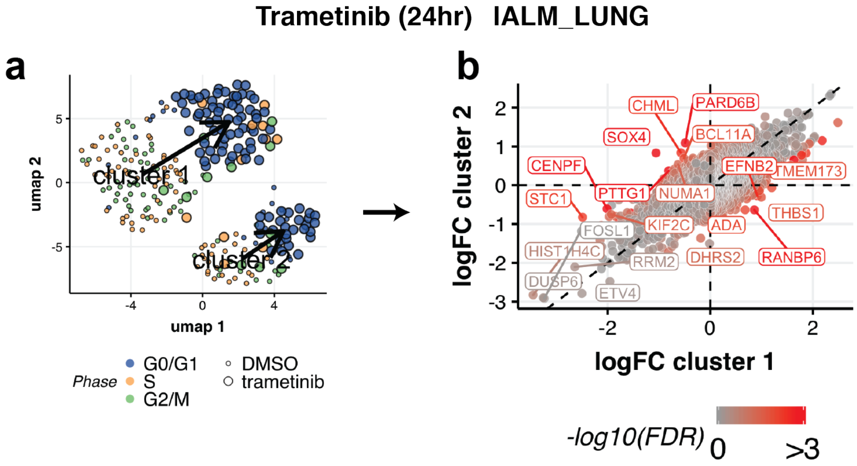

Supplementary Fig. 15: Single-cell RNA-seq allows analysis of perturbation responses in heterogeneous populations.

As an example demonstrating the ability of MIX-Seq to resolve differential transcriptional responses among distinct sub-populations of cells, we identified two distinct sub-populations of IALM cells (present in the baseline data), and measured the transcriptional response of each sub-population to trametinib. **a**) UMAP plot showing response of each sub-population to trametinib treatment (24 hours post-treatment). Fill color depicts inferred cell-cycle phase, and dot size depicts treatment condition. **b)** Comparison of the average trametinib response for cells from the two IALM clusters shown in **a**. Dot color represents the significance of the difference between trametinib responses of the two sub-populations. Differential expression analysis was performed using the edgeR quasi-likelihood approach (Lun et al. 2016) as used in (Soneson and Robinson 2018).

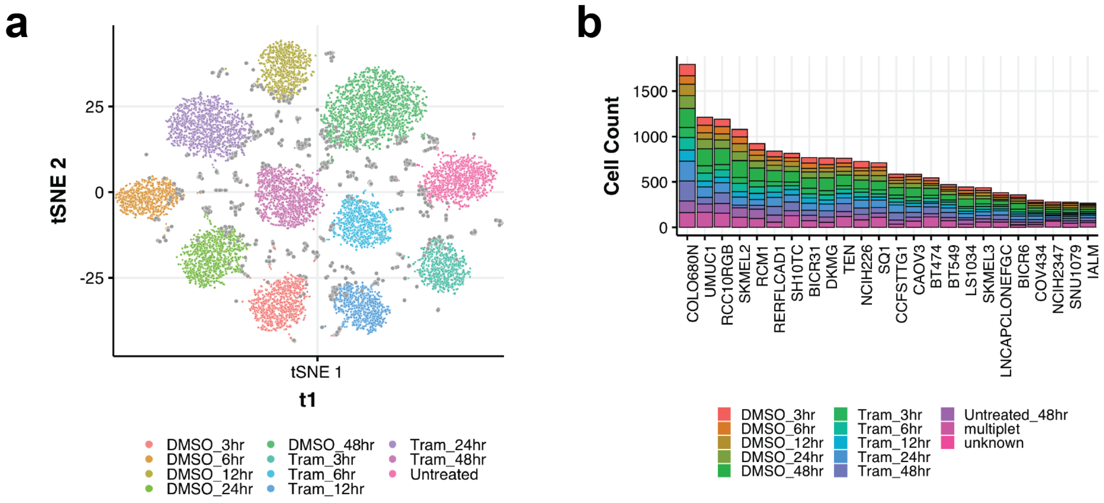

Supplementary Fig. 16: Cell Hashing provides efficient labeling of cells from different experimental conditions

a) t-SNE representation of hashtag read profiles across single cells from all cell lines, colored by the classified treatment condition. Read count profiles across hashtags were normalized for each cell using the centered log-ratio transformation (with a pseudocount value of 1) prior to computing the t-SNE embedding. Gray dots indicate cells classified as doublets. **b)** Histogram of cell counts by parental cell line and the inferred hashtag condition.

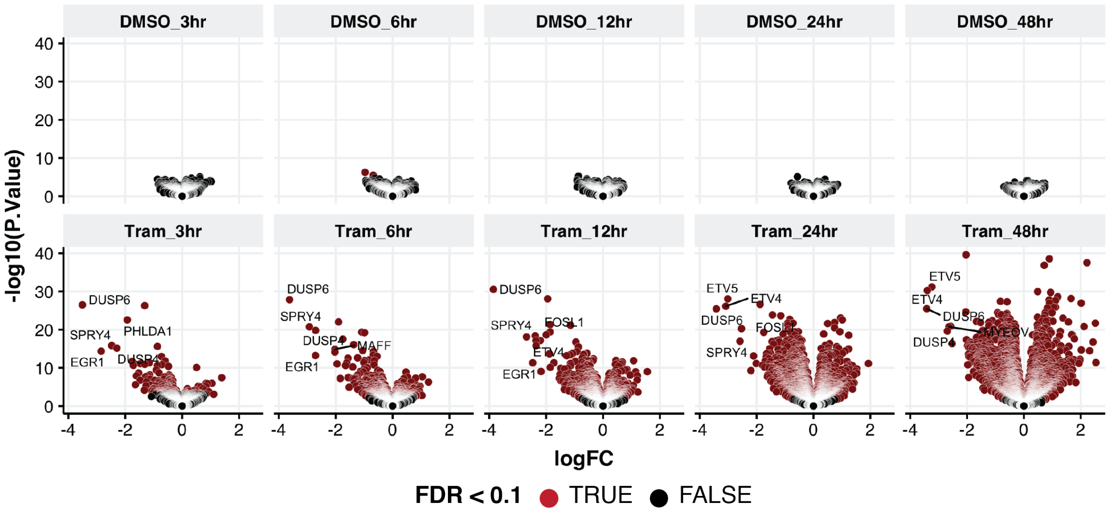

Supplementary Fig. 17: Lack of time-dependent transcriptional response following treatment with DMSO vehicle control.

Volcano plots showing the average (across cell lines) transcriptional response to each treatment condition, using untreated cells as reference. Virtually no genes showed significant changes (FDR < 0.1) in any DMSO-treated conditions, and there were no time-dependent trends apparent in the DMSO response. As a result, we combined DMSO conditions, and untreated cells, as reference for other analyses.

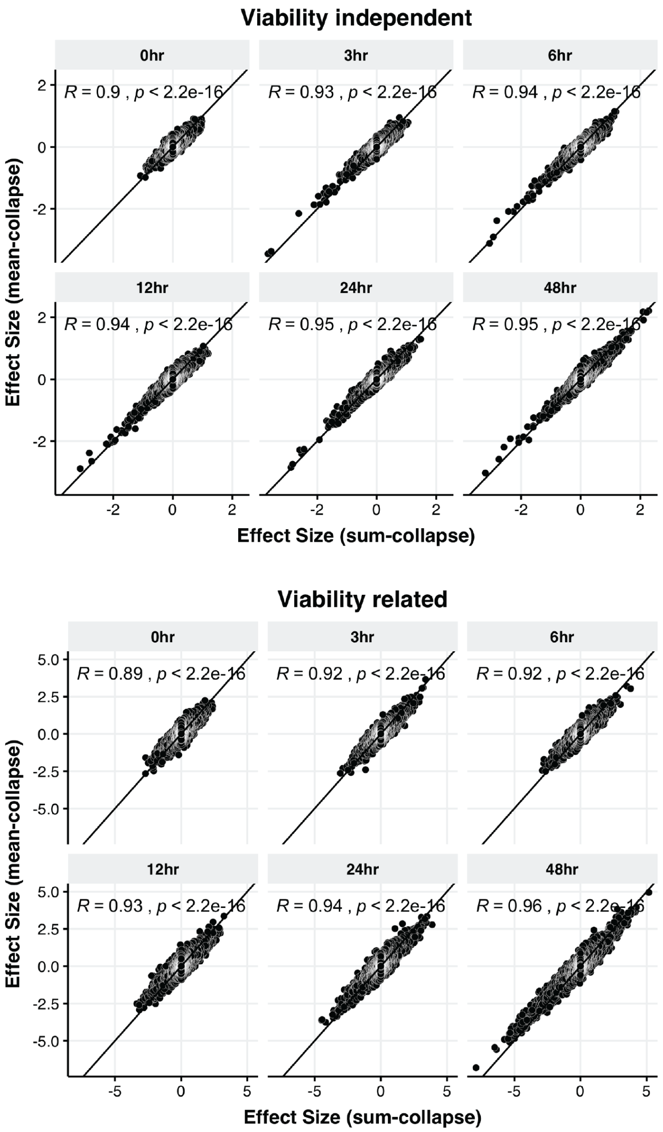

Supplementary Fig. 18: Estimation of viability-related and viability-independent response components is unbiased by cell count

To ensure that our analyses of the viability-related and -independent time-courses of trametinib responses in **Fig. 5e,f** were unbiased by the number of cells available in each cell line and post-treatment time point we performed additional tests. Specifically, we recomputed these response components after *averaging* the normalized expression profiles across cells for each cell line, rather than *summing* the read counts across cells as in **Fig. 5e,f**. Averaging the normalized expression profiles ensures that the results are unbiased by the number of available cells. Comparison of the viability-independent (*top*) and viability-related (*bottom*) trametinib response components at each post-treatment time point shows close agreement independent of the method of aggregating data across cells. Pearson correlation coefficients and associated P-values are included for each plot.

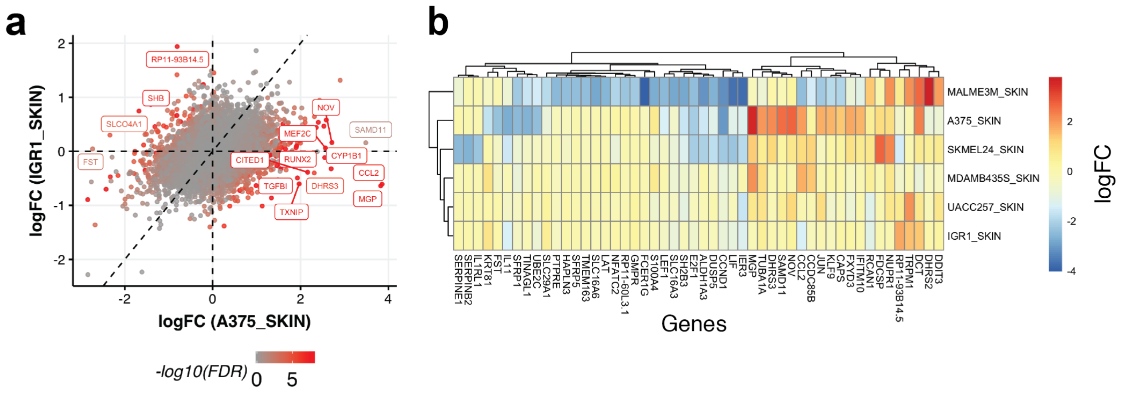

Supplementary Fig. 19: Variability of dabrafenib responses among BRAF mutant melanomas.

a) Scatterplot comparing the average response of IGR1 and A375 cells to dabrafenib treatment (24 hour post-treatment) across genes. Genes with significantly different responses between the two cell lines are indicated in red. **b)** Heatmap showing dabrafenib responses across six BRAF mutant melanoma lines for the top 50 genes with most variable responses. LFC estimates are computed with a pseudo-count parameter of 10 to stabilize estimates for low-abundance genes.

References

Calle, Y., Palomares, T., Castro, B., del Olmo, M. and Alonso-Varona, A. 2000. Removal of N-glycans from cell surface proteins induces apoptosis by reducing intracellular glutathione levels in the rhabdomyosarcoma cell line S4MH. *Biology of the Cell* 92(8–9), pp. 639–646.

Corsello, S.M., Nagari, R.T., Spangler, R.D., et al. 2020. Discovering the anticancer potential of non-oncology drugs by systematic viability profiling. *Nature Cancer.*

Iorio, F., Knijnenburg, T.A., Vis, D.J., et al. 2016. A landscape of pharmacogenomic interactions in cancer. *Cell* 166(3), pp. 740–754.

Jeganathan, S. and Lee, J.M. 2007. Binding of elongation factor eEF1A2 to phosphatidylinositol 4-kinase beta stimulates lipid kinase activity and phosphatidylinositol 4-phosphate generation. *The Journal of Biological Chemistry* 282(1), pp. 372–380.

Kang, H.M., Subramaniam, M., Targ, S., et al. 2018. Multiplexed droplet single-cell RNA-sequencing using natural genetic variation. *Nature Biotechnology* 36(1), pp. 89–94.

Law, C.W., Chen, Y., Shi, W. and Smyth, G.K. 2014. voom: Precision weights unlock linear model analysis tools for RNA-seq read counts. *Genome Biology* 15(2), p. R29.

Lun, A.T.L., Chen, Y. and Smyth, G.K. 2016. It's DE-licious: A Recipe for Differential Expression Analyses of RNA-seq Experiments Using Quasi-Likelihood Methods in edgeR. *Methods in Molecular Biology* 1418, pp. 391–416.

Ritchie, M.E., Phipson, B., Wu, D., et al. 2015. limma powers differential expression analyses for RNA-sequencing and microarray studies. *Nucleic Acids Research* 43(7), p. e47.

Soneson, C. and Robinson, M.D. 2018. Bias, robustness and scalability in single-cell differential expression analysis. *Nature Methods*.

Subramanian, A., Narayan, R., Corsello, S.M., et al. 2017. A next generation connectivity map: L1000 platform and the first 1,000,000 profiles. *BioRxiv*.
