# Supplementary material for: Multiplexed single-cell transcriptional response profiling to define cancer vulnerabilities and therapeutic mechanism of action
Source: Nat Commun. 2020 Aug 27;11:4296. doi: 10.1038/s41467-020-17440-w (PMC7453022; doi:10.1038/s41467-020-17440-w)
Supplement: Supplementary file 3 — Description of Additional Supplementary Files [file 41467_2020_17440_MOESM3_ESM.docx]

**Description of Additional Supplementary Files**

Supplementary Data 1: Provides a list of all cell lines used in this study, and the experiments in which each cell line appeared.

Supplementary Data 2: Provides a description of each experiment, including the perturbation, time point, number of cell lines, and number of cells.

Supplementary Data 3: Provides a description of the drug treatments, including the drug concentrations used.
