# Supplementary material for: Multiplexed single-cell transcriptional response profiling to define cancer vulnerabilities and therapeutic mechanism of action
Source: Nat Commun. 2020 Aug 27;11:4296. doi: 10.1038/s41467-020-17440-w (PMC7453022; doi:10.1038/s41467-020-17440-w)
Supplement: Supplementary file 7 — Reporting Summary [file 41467_2020_17440_MOESM7_ESM.pdf]

nature research

Corresponding author(s): James McFarland

Last updated by author(s): Jun 23, 2020

Reporting Summary

Nature Research wishes to improve the reproducibility of the work that we publish. This form provides structure for consistency and transparency in reporting. For further information on Nature Research policies, see Authors & Referees and the Editorial Policy Checklist .

Statistics

For all statistical analyses, confirm that the following items are present in the figure legend, table legend, main text, or Methods section.

n/a	Confirmed	
☐	☒	The exact sample size (*n*) for each experimental group/condition, given as a discrete number and unit of measurement
☒	☐	A statement on whether measurements were taken from distinct samples or whether the same sample was measured repeatedly
☐	☒	The statistical test(s) used AND whether they are one- or two-sided<br>*Only common tests should be described solely by name; describe more complex techniques in the Methods section.*
☐	☒	A description of all covariates tested
☐	☒	A description of any assumptions or corrections, such as tests of normality and adjustment for multiple comparisons
☐	☒	A full description of the statistical parameters including central tendency (e.g. means) or other basic estimates (e.g. regression coefficient) AND variation (e.g. standard deviation) or associated estimates of uncertainty (e.g. confidence intervals)
☒	☐	For null hypothesis testing, the test statistic (e.g. *F*, *t*, *r*) with confidence intervals, effect sizes, degrees of freedom and *P* value noted<br>*Give P values as exact values whenever suitable.*
☒	☐	For Bayesian analysis, information on the choice of priors and Markov chain Monte Carlo settings
☒	☐	For hierarchical and complex designs, identification of the appropriate level for tests and full reporting of outcomes
☐	☒	Estimates of effect sizes (e.g. Cohen's *d*, Pearson's *r*), indicating how they were calculated

Our web collection on statistics for biologists contains articles on many of the points above.

Software and code

Policy information about availability of computer code

Data collection	No specialized software was used for data collection
Data analysis	10x single-cell RNA-Sequencing data were processed using Cell Ranger software (v2 and v3). Classification of single cell identities (based on SNPs and gene expression profiles) was done using custom analysis code available at: https://github.com/broadinstitute/single_cell_classification. This analysis utilized the following R packages: glmnet v3.0.2, mclust v5.4.5, DropletUtils v1.6.1, vcfR v.1.9.0<br><br>Code to perform the remaining analysis in the paper, as well as generating the figures, is available at https://github.com/broadinstitute/mix_seq_ms. This analysis used the following R packages: Seurat v3.1.4, ranger v0.12.1, limma v3.83.3, edgeR v3.24.3, GSEABase v1.44.0, piano v1.22.0

For manuscripts utilizing custom algorithms or software that are central to the research but not yet described in published literature, software must be made available to editors/reviewers. We strongly encourage code deposition in a community repository (e.g. GitHub). See the Nature Research guidelines for submitting code & software for further information.

Data

Policy information about availability of data

All manuscripts must include a data availability statement. This statement should provide the following information, where applicable:

- Accession codes, unique identifiers, or web links for publicly available datasets
- A list of figures that have associated raw data
- A description of any restrictions on data availability

All data reported in this manuscript, including single-cell RNA-sequencing data, drug sensitivity measures, and other cell line features used in the analysis, can be accessed at https://figshare.com/s/139f64b495dea9d88c70. Additional data used in the analysis are also publicly available. Cell line ‘omics and CRISPR genetic dependency data are taken from the 19Q3 DepMap dataset available at depmap.org or from figshare at [https://figshare.com/articles/

DepMap_19Q3_Public/9201770/2]. The cell line drug sensitivity data was taken from the Sanger GDSC dataset 4,7, which is available for download from depmap.org or https://www.cancerrxgene.org/, and data generated using the PRISM multiplexed drug screening platform 17,20, which is available for download from depmap.org. The L1000 gene expression signatures were taken from either the LINCS Phase 2 data (GEO accession GSE70138, downloaded from http://amp.pharm.mssm.edu/Slicr) or LINCS Phase 1 data (GEO accession GSE92742, downloaded from clue.io).

Field-specific reporting

Please select the one below that is the best fit for your research. If you are not sure, read the appropriate sections before making your selection.

- [x] Life sciences
- [ ] Behavioural & social sciences
- [ ] Ecological, evolutionary & environmental sciences

For a reference copy of the document with all sections, see nature.com/documents/nr-reporting-summary-flat.pdf

Life sciences study design

All studies must disclose on these points even when the disclosure is negative.

Sample size

We aimed to recover 100 or more single-cell profiles per cell line and condition in our initial perturbation-response experiments, reasoning that this would be sufficient for robustly estimating average transcriptional responses, as well as allowing analyses of population heterogeneity. The number of cell lines used in each experiment varied from 24 to 99, with larger numbers of cell lines increasing power for analyses of the variation in responses across cell lines. We apply downsampling analyses to show how both cell/cell line, and cell line sample sizes affect results.

Data exclusions

Gene expression profiles of individual cells were excluded from analysis if they did not pass quality-control measures, as described in the manuscript. These were: cells where the proportion of UMIs from mitochondrial genes was < 0.01 or > 0.25; cells identified as putative empty droplets based on clustering analysis of the SNP model fit stats across cells; cells identified as doublets using the SNP model; and cells that did not have a sufficiently high confidence in the assignment to a particular cell line.

Additionally, two experiments were excluded from analysis. One using a dose of everolimus that we determined was too low (everolimus treatment was repeated). The second treatment (with the CDK7i THZ-2-102-1) was too toxic to the cells at the dose and time point used to recover usable transcriptional profiles. These exclusion criteria were not pre-established.

Replication

Robustness of our results is facilitated by analyzing many individual cells per sample/condition, and also many cell lines per treatment. Additionally, transcriptional response to trametinib (24 hours post-treatment) were measured 3 times in separate experiments (in different cell line pools), and the results showed good agreement across experiments.

Randomization

For analyses of perturbation responses (drug and CRISPR perturbations), treatment and control responses were measured for all cell lines, and there was no need to assign samples into control or treatment groups.

Blinding

Blinding was not relevant to the study because all samples were measured in both treatment and control groups.

Reporting for specific materials, systems and methods

We require information from authors about some types of materials, experimental systems and methods used in many studies. Here, indicate whether each material, system or method listed is relevant to your study. If you are not sure if a list item applies to your research, read the appropriate section before selecting a response.

Materials & experimental systems

n/a	Involved in the study
[ ]	[x] Antibodies
[ ]	[x] Eukaryotic cell lines
[x]	[ ] Palaeontology
[x]	[ ] Animals and other organisms
[x]	[ ] Human research participants
[x]	[ ] Clinical data

Methods

n/a	Involved in the study
[x]	[ ] ChIP-seq
[x]	[ ] Flow cytometry
[x]	[ ] MRI-based neuroimaging

Antibodies

Antibodies used

For Cell Hashing experiments we used BioLegend TotalSeq™-A Hashing antibodies #4-10 and 12-15 (product codes: 394607, 394609, 394611, 394613, 394615, 394617, 394619, 394623, 394625, 394627, 394629)

Validation

BioLegend reports that they are "operating under an ISO 13485:2016 certified quality management system", and provide detailed information on their product pages

Eukaryotic cell lines

Policy information about cell lines

Cell line source(s)	Parental cell lines were obtained from the Broad-Novartis Cancer Cell Line Encyclopedia (CCLE) project (see https://portals.broadinstitute.org/ccle for original sources). The cell lines used were (this list can also be found in Supplementary Table 1): CALU6, DANG, GOS3, HS939T, HUH6, JHOS2, MFE280, OVSAHO, OVTOKO, PK59, RVH421, SKMEL30, SNU738, SNUC4, T47D, TCCSUP, TE1, TOV21G, VMRCRCZ, CL34, COLO792, HEC59, HS729, KMRC3, KYSE520, MKN7, NCIH1568, NCIH1792, NCIH1944, NCIH2087, NCIH358, NCIH727, PANC0203, PK45H, RD, SNU423, SW1990, ZR751, A204, ABC1, CAMA1, EBC1, HCC56, HEC151, HOS, HT55, LS180, MDAMB436, MKN45, NCIH1373, NCIH1435, NCIH292, OUMS23, RERFLCAI, RT4, SIMA, SKES1, SKMES1, SNU1196, SNU449, TE9, 2313287, BCPAP, BHY, BICR56, CAS1, CCK81, DETROIT562, EFE184, EKVX, HCC1428, HDQP1, HUPT3, HUPT4, MDAMB361, NCIH2110, NCIH2170, NCIH650, OVKATE, PECAPJ49, RERFLCKJ, SNU81, SW1088, SW1463, BICR18, C32, COLO741, HCC38, HEC108, HEC251, HT1197, HT1376, JHH7, KPNSI9S, MCF7, MFE319, NCIH2196, NCIH2452, NCIH446, NCIH747, SKNAS, SNU308, SNU899, SW1116, SW1271, TM31, TYKNU, WM88, 8305C, ASPC1, BICR16, CAKI2, HCC366, HEC6, HUH28, IM95, KALS1, NCIH2073, NCIH2228, OAW28, ONCODG1, OVCAR4, SCC25, SKMEL5, SNU46, SU8686, TE10, U118MG, 8MGBA, A101D, A172, A673, A704, ACHN, AN3CA, BC3C, BFTC905, BHT101, CADOES1, CAKI1, CAL27, CAL51, CAL78, CAPAN2, CFPAC1, COLO679, COLO783, COLO800, CORL105, COV362, DBTRG05MG, DU145, ECGI10, EFO21, EN, ESS1, EWS502, FADU, G292CLONEA141B1, G402, GCT, GMS10, HARA, HCC1359, HCC1438, HCC15, HCC1588, HCC1937, HCC4006, HCC44, HCC78, HCC95, HEC265, HS618T, HS944T, HSC2, HSC3, HT144, HUH7, IPC298, JHH1, JHH4, JHUEM2, JIMT1, JMSU1, K029AX, KELLY, KMRC1, KMRC20, KNS62, KP3, KURAMOCHI, KYSE140, KYSE150, KYSE180, KYSE30, KYSE410, LCLC103H, LN18, LOUNH91, LS411N, LXF289, MDAMB175VII, MDAMB231, MELHO, MELJUSO, MEWO, MFE296, MG63, MHHES1, MKN1, MKN74, MON, MPP89, NB1, NCIH1623, NCIH1651, NCIH1838, NCIH2023, NCIH2030, NCIH2052, NCIH2122, NCIH2172, NCIH520, NCIH596, NCIH661, NUGC4, OC314, OE19, OE21, OE33, OV56, OV7, OVCAR8, PANC0327, PANC0813, PANC1005, PATU8902, PC14, PECAPJ15, PECAPJ41CLONED2, PK1, PLCPRF5, PSN1, RERFLCMS, RKN, RL952, RMGI, RT112, SCABER, SF126, SF539, SH4, SKHEP1, SKLU1, SKNBE2, SKNEP1, SKOV3, SNGM, SNU1066, SNU213, SNU398, SNU407, SNU466, SNU601, SNU668, SNU719, SNU869, SNU886, SNUC5, SW1353, SW1573, SW1710, SW780, SW837, T3M10, T98G, TE4, TE5, TM87, TT, TDOTT, TT2609C02, U251MG, U2OS, UACC62, UBLC1, VMRCRCW, WM793, WM983B, YAPC, YD8, YH13, YKG1
Authentication	Single cells profiled in this experiment were matched to parental cell lines in the CCLE based on their SNP profiles, as described in the manuscript. We note that some cell lines in the PRISM collection have STR profiles that do not match those reported by vendors and in the literature, as reported in (Corsello et al., 2019, bioRxiv, https://doi.org/10.1101/730119). STR fingerprinting was performed by Genetica using the PowerPlex 16 HS system (Promega).
Mycoplasma contamination	Cell line pools were validated as mycoplasma free prior to initiating each experiment
Commonly misidentified lines<br>(See ICLAC register)	MDAMB435S (ICLAC-00565) has been listed in ICLAC as a misidentified breast cell line. The annotation we used has been updated to reflect that it is in fact a melanoma cell line.
